# Gut microbiome profiles may be related to atypical antipsychotic associated overweight in Asian children with psychiatric disorder: a preliminary study

**DOI:** 10.3389/fcimb.2023.1124846

**Published:** 2023-05-03

**Authors:** Shao-rui Hao, Yuan-yue Zhou, Xue Zhang, Hai-yin Jiang

**Affiliations:** ^1^ Collaborative Innovation Center for Diagnosis and Treatment of Infectious Diseases, State Key Laboratory for Diagnosis and Treatment of Infectious Diseases, The First Affiliated Hospital, School of Medicine, Zhejiang University, Hangzhou, Zhejiang, China; ^2^ Department of Medical Psychology, The First Affiliated Hospital, Hainan Medical University, Haikou, Hainan, China; ^3^ Department of Child Psychiatry, Hangzhou Seventh People’s Hospital, Hangzhou, Zhejiang, China; ^4^ Department of Infectious Diseases, The Affiliated Hangzhou First People’s Hospital, School of Medicine, Zhejiang University, Hangzhou, China

**Keywords:** obesity, metabolic, microbiome, depressive, antipsychotic agents

## Abstract

**Objective:**

Atypical antipsychotics (APs) modify the gut microbiome, and weight gain in response to AP could be mediated by the gut microbiome. Thus, the present study aimed to explore the changes in the gut bacterial microbiome in AP-exposed children with obesity.

**Methods:**

To rule out the confounder of AP indication, the gut bacterial microbiome was compared between healthy controls (Con) and AP-exposed individuals with overweight (APO) or normal weight (APN). Fifty-seven AP-treated outpatients (21 APO and 36 APN) and 25 Con were included in this cross-sectional microbiota study.

**Results:**

AP users, regardless of body mass index, exhibited decreased microbial richness and diversity and a distinct metagenomic composition compared to the Con. Although no differences in the microbiota structure were observed between APO and APN groups, the APO group was characterised by a higher abundance of *Megamonas* and *Lachnospira*. Additionally, the differences in the microbial functions were observed between APO and APN groups.

**Conclusions:**

The gut bacterial microbiota of APO children revealed taxonomic and functional differences compared to Con and APN. Further studies are needed to verify these findings and to explore the temporal and causal relationships between these variables.

## Introduction

Atypical antipsychotic (AP) drugs are widely prescribed to treat schizophrenia and bipolar disorder. They are also often prescribed to children and adolescents with an eating disorder, conduct disorder, autism and other psychiatric conditions (Smogur et al., 2022). A notable increase in the number of children treated with AP medication has been observed (Hunter et al., 2022). Of particular concern is the unwanted side effects of APs, such as weight gain, and the risk is higher in children than in adults (Florentia et al., 2023). It has been reported that nearly 50% of children treated with APs develop overweight (Patel et al., 2007), which may lead to discontinuation of AP treatment with the risk of relapse and hospitalisation. Additionally, weight gain may eventually lead to overweight, obesity, metabolic syndrome, and type 2 diabetes (Lambert et al., 2006), and thereby increase the risk of cardiovascular illness (Koponen et al., 2022). Although several demographic and clinical features associated with this side effect have been reported (Martínez-Ortega et al., 2013), there is a marked inter-individual variation in AP-induced weight gain.

Increasing evidence demonstrates a vital role of the gut microbiota in the development of overweight and obesity. Studies carried out in mice have verified the causal relationship between the gut microbiota and weight gain through faecal transplantation from obese donors (Ussar et al., 2015). Epidemiological evidence shows that disrupting the gut microbiome with antibiotics in early life predicts weight gain or obesity in the short and long term (Meng et al., 2021). An emerging body of studies have linked the gut microbiome with AP-associated weight gain, but inconsistent findings have been reported. Yuan et al. demonstrated significant changes in the abundance of microbial species following 24 weeks of risperidone treatment in drug-naive schizophrenic patients (Yuan et al., 2018). Additionally, Flower et al. revealed that patients treated with AP were characterised by the gut microbiota of obesity compared to untreated patients (Flowers et al., 2017). Bahr et al. found the metabolic and microbiota changes during risperidone treatment of five male psychiatric children. Their findings reported a gradual increase in the Firmicutes to Bacteroidetes ratio, which is correlated with an increase in body mass index (BMI) (Bahr et al., 2015). Studies have demonstrated antipsychotic-associated changes in the stool microbiome, but most based on samples obtained from adult patients. Therefore, the role of the microbiome in AP-induced overweight should be evaluated in paediatric psychiatric patients. Our aims were to evaluate the effect of chronic AP use on the relative abundance of gut microbiota taxa in psychiatric children and healthy controls in a cross-sectional study.

## Methods

### Study population

This was conducted as part of a larger prospective cohort study, which investigated the role of the gut microbiome in children and adolescents with mental disorders. Enrolment began in March 2019, as described in the Chinese Clinical Trial Registry (Registration number: ChiCTR1900024396). Patients or their parents were informed of the study and consented to participate. This study was approved by the First Affiliated Hospital of Zhejiang University (Reference number: 20191034), and all procedures were carried out following the Declaration of Helsinki.

Patients aged between 8 to 16 years who presented at the Seventh People’s Hospital of Hangzhou were recruited between March 2019 and March 2020. Two experienced psychiatrists used the International Classification of Diseases (ICD)-10 and Mini-International Neuropsychiatric Interview to verify the diagnosis (Sheehan et al., 1998). In addition, a trained graduate student interviewed the patients or their parents with a structured questionnaire about drug use within 30 days of admission, using a series of colour pictures of the agents of interest (antibiotics, acid-suppressing drugs, antipsychotics, antidepressants, mood stabilisers, probiotics, prebiotics, and synbiotics).

The exclusion criteria were established as following: psychosis; anorexia; malnutrition; inflammatory bowel disease; celiac disease; irritable bowel syndrome; diabetes mellitus; liver diseases; drug or alcohol abuse; use of antibiotics, probiotics, prebiotics, or synbiotics in the month before collection of the fecal sample.

### Study design

Stool was collected to determine the composition of the microbiota in a group of paediatric AP-treated inpatients who provided informed consent. AP use was defined as continuous use of an AP daily for at least 6 months before stool collection. We also enrolled age- and gender-matched controls without mental disease and not on APs. According to the BMI (overweight was defined as BMI≥24) and AP exposure, all participants were classified into three groups: (1) AP with overweight (APO), (2) AP without overweight (APN), and (3) healthy control without overweight (Con). Only patients in remission were included to rule out the influence of psychiatric symptoms on the gut microbiota.

### Faecal sample processing

The stool samples from a cross-sectional study were collected in sterile plastic cups as soon as they went to the outpatient clinic. The second sample was collected at home and stored at −20°C. All samples were shipped in dry ice to our laboratory and stored at −80°C until DNA extraction.

DNA was extracted by bead beating on a FastPrep instrument (MP Biomedicals, Santa Ana, California) followed by genomic DNA extraction with a FastDNA kit (MP Biomedicals, Santa Ana, CA, USA). The purity and concentration of the metagenomic DNA was measured using a NanoDrop ND-2000 spectrophotometer, and integrity and size were assessed using 1.0% agarose gel electrophoresis on gels containing 0.5 mg/mL ethidium bromide. The V1-V3 region of the bacterial 16S rRNA gene was amplified using a polymerase chain reaction (PCR) procedure and the barcode primer set 338F ACTCCTACGGGAGGCAGCAG and 806R GGACTACHVGGGTWTCTAAT. The DNA products were purified by gel extraction and quantified using QuantiFluor-ST (Promega, Madison, WI, USA). The purified amplicons were pooled in equimolar concentrations and sequenced using the Illumina MiSeq platform with a TruSeqTM DNA Sample Prep Kit (Illumina, San Diego, CA, USA).

### Sequence and statistical analysis

The sequencing reads were processed using Quantitative Insights into Microbial Ecology (QIIME) software, preliminary quality control steps were performed as described in a previous study, and chimera sequences were removed with ChimeraSlayer. The remaining effective sequences were binned into operational taxonomic units (OTUs) with a cutoff of 97% identity to determine alpha diversity (Shannon and sobs), richness (ACE and Chao1). Beta diversity was estimated by computing weighted UniFrac values, and visualized using a principal coordinate analysis. A linear discriminant analysis effect size (LEfSe) procedure was used to identify biomarkers of microbiomes in the fecal samples at genus taxonomic ranks; an effect size threshold (on the logarithmic LDA score) of 2 was used (Segata et al., 2011). Metagenomic functional composition prediction analysis for all samples based on 16S data in the latest Kyoto Encyclopedia of Genes and Genomes (KEGG) database was performed using the PICRUSt pipeline. Using the linear discriminant analysis (LDA) effect size measurements (LEfSe) method, we further selected the microbiome features and relative abundances of the predicted KEGG pathways significantly associated with depression. In the present study, an effect size threshold (based on the logarithmic LDA score) of 2 was used. Multivariate Association with Linear Models algorithm (MaAsLin) was used for association testing of the covariates versus the abundance of microbial taxa with default parameters, to deconfound the effects of gender, type of AP, AP dose, and other psychoactive drug use. We also performed power and sample number simulations using the pwr (version 1.1-3) R package and base R functions. If effect size is set to 0.6, we would need at least 27 samples in each group to power the full analysis. All statistical analyses were performed using the SPSS data analysis program (version 21.0) and R packages (V.2.15.3) (McMurdie and Holmes, 2013). Therefore, Independent paired-samples t-tests and Mann–Whitney U-tests were applied to assess continuous variables, and Chi-square or Fisher’s exact tests were used to evaluate categorical variables, depending on assumption validity. P-values <0.05 were considered to indicate statistical significance.

## Results

### Study population

The study included 57 AP-treated inpatients and 25 controls. No significant differences in age, gender or psychiatric symptoms were observed between the groups. The BMI index was significant high in the APO group than in the APO and Con groups; however, there was no difference between APN and Con groups. The dose of sertraline was 25 mg daily during the first week, and thereafter 50-100 mg once daily. Of the 57 psychiatric patients, 40 were on an AP for an average of 12 months for depression, 9 for anxiety, 6 for attention deficit hyperactivity disorder (ADHD) and 2 for conduct disorder. The leading APs used were risperidone (n = 29) followed by quetiapine (n = 16), aripiprazole (n = 8) and olanzapine (n = 3). The dose of the individual AP was as following: risperidone (0.5-1mg), quetiapine (25-50mg), aripiprazole (2.5mg) and olanzapine (2.5mg). The characteristics of participants among the three groups are presented in **Table 1**.

**Table 1:** Clinical characteristics of study subjects in cross-sectional microbiota study.

Parameters	APO (n=21)	APN (n=36)	Con (n=25)	P
Proportion of Females, n (%)	13 (62%)	20 (55%)	13 (52%)	> 0.05
Age (years; means ± SD)	13.2 ± 3.1	12.9 ± 2.8	13 ± 3.2	> 0.05
BMI (means ± SD)	26.2 ± 7.6	19.3 ± 5.3	18.9 ± 4.9	< 0.05^#^
Psychiatric characteristics				
Depressive disorder, n (%)	15 (71.4%)	25 (69.4%)	0	> 0.05*
Anxiety disorder, n (%)	3 (14.3%)	6 (16.7%)	0	> 0.05*
ADHD, n (%)	2 (9.5%)	4 (15.4%)	0	> 0.05*
Conduct disorder, n (%)	1 (4.8%)	1 (2.8%)	0	> 0.05*
AP treatment duration, months	12.4 ± 6.3	11.3 ± 6.1	0	> 0.05*
Type of AP				
Risperidone, n (%)	10 (47.6%)	19 (52.8%)	0	> 0.05*
Quetiapine, n (%)	6 (28.6%)	10 (27.8%)	0	> 0.05*
Aripiprazole, n (%)	3 (14.3%)	5 (13.9%)	0	> 0.05*
Olanzapine, n (%)	1 (4.8%)	2 (5.6%)	0	> 0.05*
Other psychopharmacology				
Sertraline, n (%)	18 (85.8%)	31 (86.1%)	0	> 0.05*
Psychostimulants, n (%)	3 (14.2%)	5 (3.9%)	0	> 0.05*

*APO compared with APN; ^#^ APO compared with APN or Con; ADHD, attention deficit hyperactivity disorder; APO, atypical antipsychotic with overweight; APN, atypical antipsychotic without overweight; BMI, body mass index; Con, healthy control; SD, standard deviation; SSRIs, selective serotonin reuptake inhibitors.

### Altered gut microbiota structure in participants with AP-associated weight gain

**Figure 1:**
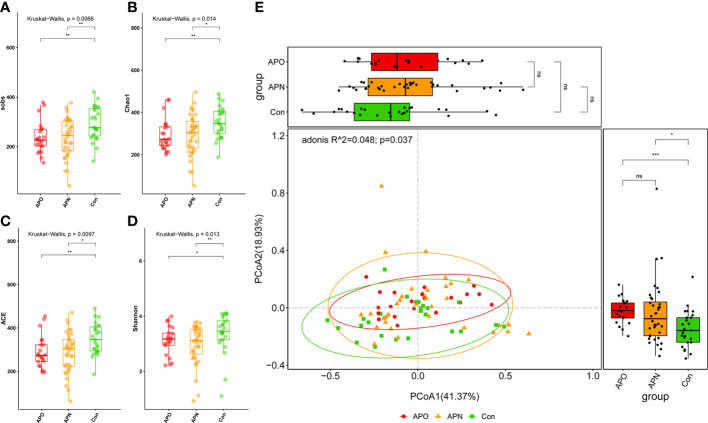
Comparison of the sobs **(A)**, Chao1 **(B)**, ACE **(C)**, and Shannon **(D)** index among three groups; PCoA of weighted UniFrac distance matrix analysis demonstrated that the bacterial microbiome composition of AP-exposed individuals clustered separately from Con **(E)**. *, p < 0.05, **, p < 0.01, ***, p < 0.001, and ns, p > 0.05.

We analysed the bacterial fraction of the microbiota using high-throughput sequencing of the bacterial 16S rRNA gene. We obtained 3,105,236 high-quality sequences from 82 faecal samples, with an average of 37,868 reads aligned per sample. A variety of indices were used to evaluate α-diversity, and we observed a decrease in these α-diversity indices in the order of the control (Con), AP-exposed normal weight (APN) and AP-exposed overweight (APO) groups. Furthermore, α-diversity was significantly lower in the APN and APO groups than in the Con group (**Figures 1A–D**). Principal coordinates analysis (PCoA) based on weighted UniFrac separated the APO and APN groups from the Con group; however, the weighted UniFrac-based PCoA indicated that the overall microbial composition of APO did not deviate from that of APN (**Figure 1E**).

### Taxonomic alterations of gut microbiota in AP-associated weight gain

**Figure 2:**
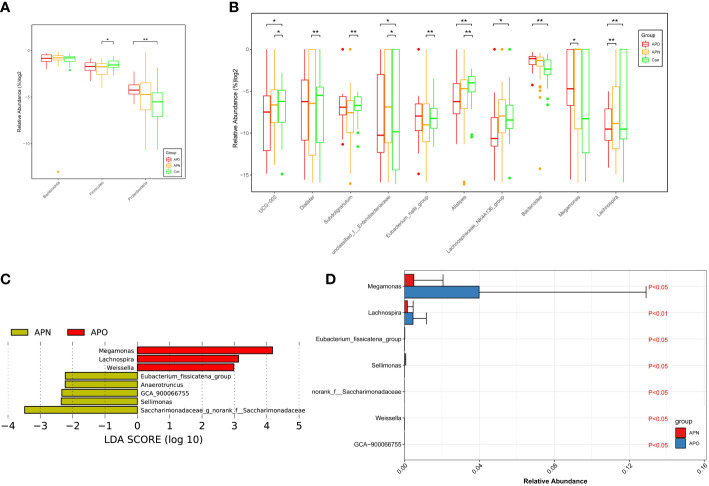
Comparison of relative abundance at the bacterial phylum **(A)** and genus **(B)** levels among three groups; APO-enriched taxa are indicated with a positive LDA score (Red), and taxa enriched in APN have a negative score (Orange). Only taxa meeting an LDA significant threshold > 2 are shown **(C)**. Genera remained associated with AP-associated overweight when using MaAsLin method adjusting for gender, type of AP, AP dose, and other psychoactive drug use. LDA effect size analysis revealed that the relative abundance of 7 genera was significantly different between APO and APN group **(D)**. *, p < 0.05, **, p < 0.01.

The analysis of phylotypes indicated that Bacteroidetes, Firmicutes and Proteobacteria were the dominant taxa in the participants. To define the compositional differences between the three groups predicted by the α-diversity analysis, the relative abundances (> 1% of the total sequences in either group) of microbes at different taxonomic levels were computed. Firmicutes spp. decreased significantly in APN patients, whereas Proteobacteria spp. were over-represented in APO patients, compared with Con (**Figure 2A**). The abundances of the ten bacterial genera were different among the three groups (**Figure 2B**). The relative abundance of *Alistipes* and UCG-002 decreased in the order of the Con, APN and APO groups, whereas the relative abundances of *Bacteroides* and *Lachnospira* decreased in the order of the APO, APN and Con groups. *Dialister*, *Subdoligranulum* and *Eubacterium_hallii*_group decreased in APN patients compared with the Con. Of the remaining taxa, *Megamonas* was enriched in APO compared to APN and *Lachnospiraceae_NK4A136_group* decreased in APO compared to Con.

A metagenomic analysis using the linear discriminant analysis effect size (LEfSe) approach was applied to identify the key phylotypes responsible for the differences between APO and APN patients. *Megamonas*, *Lachnospira* and *Welssella*, which were abundant in APO patients, were the key phylotypes that contributed to the differences in the intestinal microbiota composition between APO and APN patients (**Figure 2C**). To deconfound the effects of gender, type of AP, AP dose, and other psychoactive drug use, MaAsLin approach was applied to identify the key phylotypes responsible for the difference between APO and APN patients. *Megamonas*, *Lachnospira, Eubacterium_fissicatena_group*, *Sellimonas*, *Weissella*, *GCA−900066755* contributed to the difference between the intestinal microbiota of APO and APN (**Figure 2D**).

### Functional alterations in AP-associated weight gain

**Figure 3:**
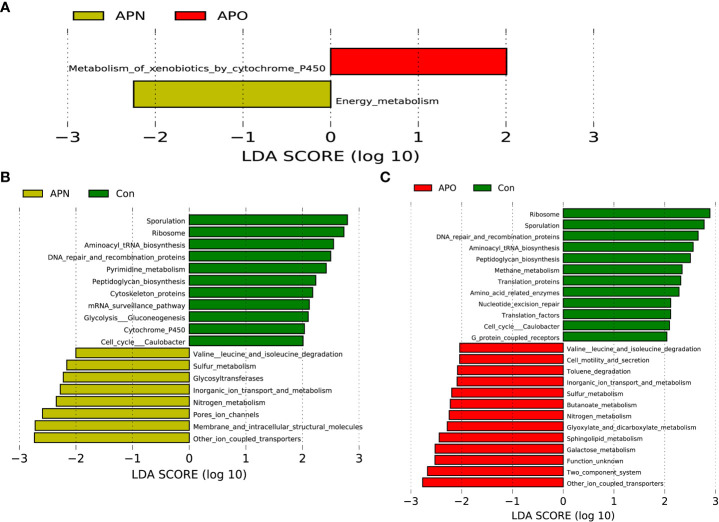
PICRUSt-based examination of the bacterial microbiome of the APO and APN groups **(A)**, APN and Con **(B)**, and APO and Con **(C)**. Comparisons between the groups for each KEGG functional category (level 3) are shown by percentage.

The functional composition of the fecal bacterial microbiome was predicted using PICRUSt, and then LEfSe was used to explore the derived KEGG pathways that were differentially expressed between APO and APN groups. Notably, in KEGG pathway level 3, metabolism of xenobiotics by cytochrome P450 was over-represented in the APO group, whereas energy metabolism was over-represented in the APN group (**Figure 3A**). Specifically, 11 pathways in level 3, including the sporulation and ribosome, and aminoacyl tRNA biosynthesis, increased significantly in Con-associated microbiota, while 8 other pathways, including other ion coupled transporters and membrane and intracellular structure molecules, increased prominently in the APN-associated microbiota (**Figure 3B**). Finally, 12 pathways in level 3, including the sporulation and ribosome, and DNA repair and recomnination proteins, increased significantly in Con-associated microbiota, while 13 other pathways, including other ion coupled transporters and two component system, increased prominently in the APO-associated microbiota (**Figure 3C**).

## Discussion

Our study is the first to evaluate the role of the gut microbiota in AP-associated overweight based on a paediatric population from South China using gene sequencing methods. The findings of this study support the previous idea that AP-associated overweight is associated with changes in the composition and function of the gut microbiota.

In a first step, we observed lower microbial α-diversity in AP-exposed individuals independent of the BMI index, indicating that a reduction in bacterial richness is associated with psychiatric (Jiang et al., 2018; Jiang et al., 2020) and metabolic diseases (de Cuevillas et al., 2022; Zheng et al., 2022). These results were consistent with Flowers et al. (2017), who used 16S ribosomal sequences from 117 adult bipolar disorder patients (49 AP treated and 68 non-AP treated). However, we did not identify differences in α-diversity between the APN and APO groups. Only one study by Bahr et al. (2015) evaluated the effect of APs on microbiota in children with ADHD and found greater α-diversity after chronic risperidone treatment. The possible reasons for this discrepancy could be that most of the participants in this study were diagnosed with an affective disorder, in whom the α-diversity of the gut microbiota was lower than in healthy individuals. In contrast, Bahr et al. recruited ADHD children without AP treatment as a control group. Their bacterial diversities may have differed from ours because we used healthy subjects as a control group. Finally, as bacterial diversity changes with BMI, the inconsistent results may be explained by differences in BMI because children with obesity accounted for a small proportion of their chronic risperidone treatment group.

Another major finding of this study was the changes in the abundances of ten genera. In particular, the relative abundance of *Alistipes* decreased in the order of the Con, APN and APO groups. Our previous study reported a reduction of *Alistipes* in children with depression (Jiang et al., 2020). *Alistipes* spp. are indole positive (Song et al., 2006), which could influence tryptophan availability and serotonin metabolism. Longstanding evidence exists for an association between increased serotonin levels and satiety (Voigt and Fink, 2015); thus, a reduced abundance of *Alistipes* could contribute to AP-associated obesity through indirect regulation of appetite. A lower abundance of *Alistipes* has also been observed in children and adults with obesity (Garcia-Ribera et al., 2020; Zeng et al., 2021); thus, further research is needed to clarify the role of this genus in the serotonergic system of subjects with AP-associated obesity. The LEfSe analysis determined that APO patients were characterised by enrichment with *Megamonas* and *Lachnospira* compared to APN patients. *Megamonas* (family Acidaminococcaceae) principally contributed to the increase in Proteobacteria in the APO group. Although no reports are available about the relationship between *Megamonas* and APs, an increase in *Megamonas* is assumed to be associated with metabolic diseases, such as obesity and diabetes (Chen et al., 2020; Palmas et al., 2021; Shang et al., 2021). *Megamonas* is negatively correlated with the level of physical activity (Palmas et al., 2021), and we speculate that the relative abundance of this genus in APO individuals at least partly was involved in AP-associated sedation. *Lachnospira* (family Lachnospiraceae) is known to be involved in various diseases. The enrichment of *Lachnospira* in the APO group also agreed with previous research using pyrosequenced faecal samples, in which Eastern Chinese children with obesity exhibited significant faecal overgrowth of *Lachnospira*. In another study, *Lachnospira* was prevalent in the faecal samples from patients with bipolar disorder and obesity (Flowers et al., 2017). An extensive body of data demonstrates that disrupted circadian rhythms in psychiatric disorders are linked to metabolic imbalance. Carasso et al. identified *Lachnospira* as a biomarker of the late chronotype, whose energy consumption is expected to be delayed (Carasso et al., 2021). In a recent study on human microbiota and eating behaviour during the day, a higher abundance of *Lachnospira* was found when a greater percentage of energy was consumed after 2 PM (Kaczmarek et al., 2017).

In addition to the overall changes in microbial profiles, the present study also assessed functional alterations between APO and APN groups. Cytochrome P450 contributes significantly to AP metabolism, which exists in liver and gut in human (Bousman et al., 2021). The metabolism of xenobiotics by cytochrome P450 of gut microbiota was overexpressed in APO group, implying the higher concentration of AP in the child’s gut and blood. However, previous studies demonstrated that plasma concentration of AP was positively associated with AP-associated with weight gain (Lane et al., 2006; Piatkov et al., 2017). Furthermore, dysfunction in energy metabolism has been observed in the fecal microbiotas of APO group, supporting the hypothesis that the effects of microbiota dysbiosis on AP-associated weight gain could occur through absorption and metabolism of nutrients in the gut.

The main limitation of the present study is the small sample size, and the results should be confirmed in a validation cohort. However, the findings provide important preliminary information, as this study ruled out the confounder by AP indication, which may affected the gut microbiota. Second, the varied AP use among the participants may have affected the accuracy of weight gain. However, the dose used for our patients was much lower than that used for psychotic disorders, and thus the key functional bacteria in AP-associated overweight identified in our study may be more sensitive and predictive than those in studies on psychotic disorders. Although drug use was adjusted in our analysis, the confounding effect of medications was probably not thoroughly assessed. We simply treated covariates of other psychoactive drug use as dichotomous without considering the dose effect, which may distort their impacts on gut microbial composition. Third, our study lacked information on the diet, which may lead to attenuated association results. The changes in dietary habits induced by AP use may place them in a dramatically different nutrient environment, which could also contribute to differences in the gut microbiota. Fourth, emerging data indicate that gut microbiota is sensitive to modulation by physical activity. Activity pattern and basal metabolism was not evaluated in this study, which may influence the accuracy of our findings. Finally, part of included women might already start sexual development and thus the lack of such information may impair the accuracy of our findings.

## Conclusion

In conclusion, this study provides evidence for a different microbiota profile and function in paediatric patients with AP-associated overweight compared to those without AP-associated obesity and healthy controls. Future studies involving larger cohorts with metagenomic or metabolomics analyses are needed to further elucidate the impact of the bacterial microbiota on the development of AP-associated overweight.

## Data availability statement

The data presented in the study are deposited in the NCBI repository, accession number SRP316309.

## Ethics statement

The studies involving human participants were reviewed and approved by the First Affiliated Hospital of Zhejiang University. Written informed consent to participate in this study was provided by the participants’ legal guardian/next of kin.

## Author contributions

H-YJ and S-RH conceived the study and revised the manuscript critically for important intellectual content. Y-YZ and S-RH made substantial contributions to its design, acquisition, analysis and interpretation of data. XZ participated in the design, acquisition, analysis and interpretation of data. All authors contributed to the article and approved the submitted version.
